# Identification and Validation of an Immune and Ferroptosis-Combined Index for Non–Small Cell Lung Cancer

**DOI:** 10.3389/fgene.2021.764869

**Published:** 2021-11-30

**Authors:** Yang Teng, Bo Wang, Desi Shang, Ning Yang

**Affiliations:** ^1^ Department of Oncology, The Fourth Affiliated Hospital of Harbin Medical University, Harbin, China; ^2^ Department of General Surgery in Songbei, The Fourth Affiliated Hospital of Harbin Medical University, Harbin, China; ^3^ College of Bioinformatics Science and Technology, Harbin Medical University, Harbin, China

**Keywords:** NSCLC, biomarkers, bioinformatics analysis, microenvironment non–small cell lung cancer, immune, prognosis

## Abstract

**Background:** Non–small cell lung cancer (NSCLC) is among the major health problems around the world. Reliable biomarkers for NSCLC are still needed in clinical practice. We aimed to develop a novel ferroptosis- and immune-based index for NSCLC.

**Methods:** The training and testing datasets were obtained from TCGA and GEO databases, respectively. Immune- and ferroptosis-related genes were identified and used to establish a prognostic model. Then, the prognostic and therapeutic potential of the established index was evaluated.

**Results:** Intimate interaction of immune genes with ferroptosis genes was observed. A total of 32 prognosis-related signatures were selected to develop a predictive model for NSCLC using LASSO Cox regression. Patients were classified into the high- and low-risk group based on the risk score. Patients in the low-risk group have better OS in contrast with that in the high-risk group in independent verification datasets. Besides, patients with a high risk score have shorter OS in all subgroups (T, N, and M0 subgroups) and pathological stages (stage I, II, and III). The risk score was positively associated with Immune Score, Stromal Score, and Ferroptosis Score in TCGA and GEO cohorts. A differential immune cell infiltration between the high-risk and the low-risk groups was also observed. Finally, we explored the significance of our model in tumor-related pathways, and different enrichment levels in the therapeutic pathway were observed between the high- and low-risk groups.

**Conclusion:** The present study developed an immune and ferroptosis-combined index for the prognosis of NSCLC.

## Introduction

According to cancer statistics 2020, lung cancer accounts for almost one-fourth of all cancer fatalities (Siegel et al., 2020). Non–small cell lung cancer (NSCLC) is the most frequent type of lung cancers with high morbidity along with mortality, which remains a major public health problem. Despite the current progression of NSCLC treatment, the diagnosis and treatment for NSCLC is still limited. Therefore, a better understanding of the NSCLC and identifying novel biomarkers are still needed.

The immune microenvironment constitutes an important element of cancer. For example, hepatocellular carcinoma (HCC) patients with a high immune status were associated with poorly differentiated HCC. The immune status has histological and molecular classification potential for HCC (Kurebayashi et al., 2018). The microenvironment is also considered an important integral component of NSCLC (Chae et al., 2018). Our previous study identified some immune-related genes that possessed prognostic potential for NSCLC and identified an immune gene–based risk model to predict overall survival (OS) of individuals with NSCLC (Mi et al., 2020).

Ferroptosis is a kind of iron-dependent cell death caused by unrestricted lipid peroxidation (Dixon et al., 2012). Plenty of studies have been conducted to reveal its prognostic and therapeutic potential for cancer. Ribonucleotide reductase regulatory subunit M2 (RRM2) is elevated in liver cancer tissues and cells, which could protect against ferroptosis of liver cancer cells (Yang et al., 2020). The expression of a major target of ferroptosis Xc-complex was elevated in gemcitabine-resistant pancreatic cancer cells (Tang et al., 2020), and the regulators of ferroptosis play an indispensable role in estimating the survival of individuals with pancreatic cancer (Tang et al., 2020). Increased sensitivity to ferroptosis was identified to be correlated with higher scores of CD8^+^ T cells and immune checkpoints (Tang et al., 2020). Siramesine (lysosome-disrupting agent) and lapatinib (tyrosine kinase inhibitor) synergistically induced the ferroptosis of breast cancer cells. This process was inhibited by ferrastatin-1, a potent inhibitor of ferroptosis (Ma et al., 2016). Acetaminophen and erastin exert a synergistic effect in inducing ferroptosis in NSCLC (Gai et al., 2020).

The interaction between ferroptosis and immunity has aroused the attention of researchers. The enhanced function of CD8^+^ T cells in the cancer microenvironment is a dominant mechanism of cancer immunotherapy. Wang et al. found that immunotherapy could activate CD8^+^ T cells and subsequently induce ferroptosis of cancer cells (Wang W. et al., 2019). On the contrary, ferroptosis-induced lipid metabolite release by cancer cells could modulate the function of immune cells and induce immune response (Luo et al., 2021). Therefore, the combined therapy with the ferroptosis enhancer and checkpoint blockade would be a potential cancer therapeutic approach.

However, little is known about the comprehensive status of ferroptosis and the immune response in NSCLC. Herein, we aim to analysis the association between ferroptosis and immune response in NSCLC.

## Methods and Materials

### Gene Expression Datasets of Lung Cancer

In this study, we incorporated NSCLC data from two publicly available databases. For the TCGA, the gene expression data along with the matching clinical data of lung adenocarcinoma (LUAD), as well as lung squamous cell carcinoma (LUSC) were obtained from the Genomic Data Commons (GDC, https://portal.gdc.cancer.gov/). We combined LUAD samples and LUSC samples as training cohorts, which were called “TCGA” cohort, including 1129 samples.

The gene expression microarray of NSCLC (GSE37745 and GSE50081) with matching clinical data was abstracted from Gene Expression Omnibus (GEO) (http://www.ncbi.nlm.nih.gov/geo). We integrated GSE37745 and GSE50081 samples as validation cohort, which were called “GEO” cohort, including 377 samples. Gene expression data of both the datasets were normalized using the Robust Multichip Average (RMA) approach from R package “affy”.

### Immune and Ferroptosis Gene Set

A total of 1793 immune-related genes were abstracted from immunology databases, as well as Analysis Portal (ImmPort) data resource (https://www.immport.org/home). A total of 103 ferroptosis-related genes were abstracted from the study by Luo H et al (Luo and Ma, 2021).

The protein–protein interaction (PPI) network of immune-related genes and ferroptosis-related genes was constructed and visualized using Cytoscape (https://cytoscape.org/). PPI data were obtained from the STRING (https://string-db.org/) database.

### Determination of Prognosis-Linked Signatures and Establishment of the Prognostic Model

A univariate Cox proportional regression model was adopted to select OS-linked genes from both immune and ferroptosis gene sets in TCGA training data set. Overall, 42 prognosis-linked signatures were screened with *p* < 0.05, including 38 immune genes and five ferroptosis-related genes, among which *NEDD4* was both the immune- and ferroptosis-related gene.

Next, the least absolute shrinkage and selection operator (LASSO) regression model was constructed to identify significant prognostic genes. A risk score was computed *via* considering the expression of optimized 32 signatures and correlation: Risk score = (exp gene1 * coef gene1) + (exp gene2 * coef gene2) + … + (exp gene32 * coef gene32). Patients with lung cancer were stratified into the high-risk group or low-risk group by the median of the risk score.

### Evaluation of Predictive Efficacy of Prognostic Model

Principal component analysis (PCA) was used according to the expression profile of 32 prognosis-related signatures of the prognostic model in the training (TCGA cohort) and validation sets (GEO cohort). The log-rank test was adopted to assess the difference of the survival time between high-risk patients and low-risk patients. Kaplan–Meier plots were used to present the results.

### Clinical Features Relationship Analysis for Risk Score

A one-sided Wilcoxon rank sum test was adopted to explore the difference in the risk score between patients with various clinical characteristics, including sex, patient status, lymph node, tumor recurrence, and clinical pathological stage (TNM categorization of malignant tumors) in TCGA or GEO cohorts.

A chi-square test was implemented to evaluate the relationship of the clinical pathological stage group with the risk score group in TCGA dataset (Table 1).

**TABLE 1:** Baseline features of patients in TCGA cohort.

Characteristics	Whole cohort	Low risk	High risk	*p*
TCGA cohort	(*n* = 1,057)	(*n* = 528)	(*n* = 529)	—
Gender	—	—	—	<0.001
Male	624 (59.04%)	284 (53.79%)	340 (64.27%)	—
Female	433 (40.97%)	244 (46.21%)	189 (35.72%)	—
Age	—	—	—	0.687
<65 years	416 (39.36%)	211 (39.96%)	205 (38.75%)	—
≥65 years	641 (60.64%)	317 (60.04%)	324 (61.25%)	—
T-stage	—	—	—	0.001
T1	302 (28.57%)	177 (33.52%)	125 (23.63%)	—
T2	593 (56.10%)	283 (53.60%)	310 (58.60%)	—
T3	117 (11.07%)	46 (8.71%)	71 (13.42%)	—
T4	42 (3.97%)	20 (3.79%)	22 (4.16%)	—
N-stage	—	—	—	0.107
N0	673 (63.67%)	346 (65.53%)	327 (61.81%)	—
N1	234 (22.14%)	110 (20.83%)	124 (23.44%)	—
N2	122 (11.54%)	55 (10.42%)	67 (12.67%)	—
N3	7 (0.66%)	6 (1.14%)	1 (0.19%)	—
M-stage	—	—	—	0.981
M0	781 (73.89%)	377 (71.40%)	404 (76.37%)	
M1	33 (3.12%)	16 (3.03%)	17 (3.21%)	
Stage	—	—	—	0.096
I	543 (51.37%)	292 (55.30%)	251 (47.45%)	—
II	291 (27.53%)	133 (25.19%)	158 (29.87%)	—
III	176 (16.65%)	81 (15.34%)	95 (17.96%)	—
IV	34 (3.22%)	17 (3.22%)	17 (3.21%)	—

A multivariable Cox proportional regression model was performed based on the risk score and clinical characteristics. Adjusted *p* < 0.05 signified statistical significance (Table 2).

**TABLE 2:** Multivariate Cox regression analyses of risk factors for OS.

	Adjusted hazard ratio	95% confidence interval	Adjusted *p*
Risk group (High vs low)	3.79	2.98–4.81	<0.001
T-stage			
T1 vs T2	1.29	1.01–1.64	0.044
T1 vs T3	2.00	1.43–2.80	<0.001
T1 vs T4	2.04	1.31–3.20	0.002
N-stage			
N0 vs N1	1.41	1.12–1.77	0.003
N0 vs N2	1.85	1.41–2.44	<0.001
N0 vs N3	1.85	0.46–7.47	0.385
M-stage			
M0 vs M1	2.46	1.59–3.81	<0.001
Stage			
I vs II	1.48	1.17–1.87	0.001
I vs III	2.03	1.57-2.61	<0.001
I vs IV	3.15	2.03-4.88	<0.001

### Correlation Analysis of the Risk Score With Immune Infiltration and Ferroptosis-Related Score

We explored tumor immune invasion of TCGA and GEO cohorts using the ESTIMATE (Estimation of STromal and Immune cells in MAlignant Tumor tissues using Expression data) approach by R software with the package “estimate” (Yoshihara et al., 2013). The ESTIMATE method assesses the number of stromal cells along with the invasion level of immune cells in samples. Single-sample GSEA (ssGSEA) in R package gsva was used to calculate the ferroptosis-related enrichment score which we called Ferroptosis Score for each sample based on ferroptosis-related gene sets.

The fraction of 22 tumor-invading immune cells was calculated based on CIBERSORT (https://cibersort.stanford.edu/index.php) (Newman et al., 2015) for TCGA and GEO cohorts. The one-sided Wilcoxon rank sum test was adopted to analyze the differences of infiltrative degree for immune cells, and *p* < 0.05 denoted statistical significance.

After that, we conducted expression assessment of five immune checkpoint–linked genes consisting of *PDCD1* (code *PD-1*), *BTLA and, CD274* (code *PD-L1*), along with *CTLA-4* and *CD47*. The one-sided Wilcoxon rank sum test was carried out for exploring the differences of the expression of five immune checkpoint–related genes in the high-risk group and low-risk group in TCGA and GEO cohorts, and *p* < 0.05 denoted statistical significance.

### Correlation Analysis of the Risk Score and Cancer Therapeutic Signatures

A total of 23 cancer therapeutic-predicted signature sets that we used were obtained from several studies, including “Basal_differentiation”, “EMT_differentiation”, “Immune_differentiation”, “Mismatch_repair”, “Nucleotide_excision_repair”, “p53_signaling_cascade”, “Oocyte_meiosis”, “Proteasome”, “Spliceosome”, “Pyrimidine_metabolism”, “DNA_replication” “Systemic_lupus_erythematosus”, “EGFR_ligands”, “Viral_carcinogenesis”, “FGFR3-coexpressed_genes”, “PPARG_network”, “IDH1”, “KDM6B”, “WNT-β-catenin_network”, “VEGFA”, “Hypoxia”, “Cell_cycle”, and “Progesterone-mediated_oocyte_maturation” (Motz et al., 2014; Peng et al., 2015; Sweis et al., 2016; Mariathasan et al., 2018; Spranger and Gajewski, 2018; Seiler et al., 2019; Kamoun et al., 2020; Necchi et al., 2020). ssGSEA was adopted to calculate the enrichment score of the abovementioned therapeutic signature gene sets. The one-sided Wilcoxon rank sum test was adopted to explore the differences of the enrichment score between high- and low-risk groups.

### Prediction of Immunotherapy Response

IMvigor210 was used to predict immunotherapy response (http://research-pub.gene.com/IMvigor210CoreBiologies). It is a study to investigate the anti–PD-L1 antibody atezolizumab in patients with metastatic urothelial cancer (mUCC) (Mariathasan et al., 2018). We evaluated the difference of the risk score between responsive groups [PD (progressive disease), SD (stable disease), PR (partial response), and CR (complete response)].

## Results

### Intimate Interaction Between Immune Genes and Ferroptosis-Linked Genes

**FIGURE 1:**
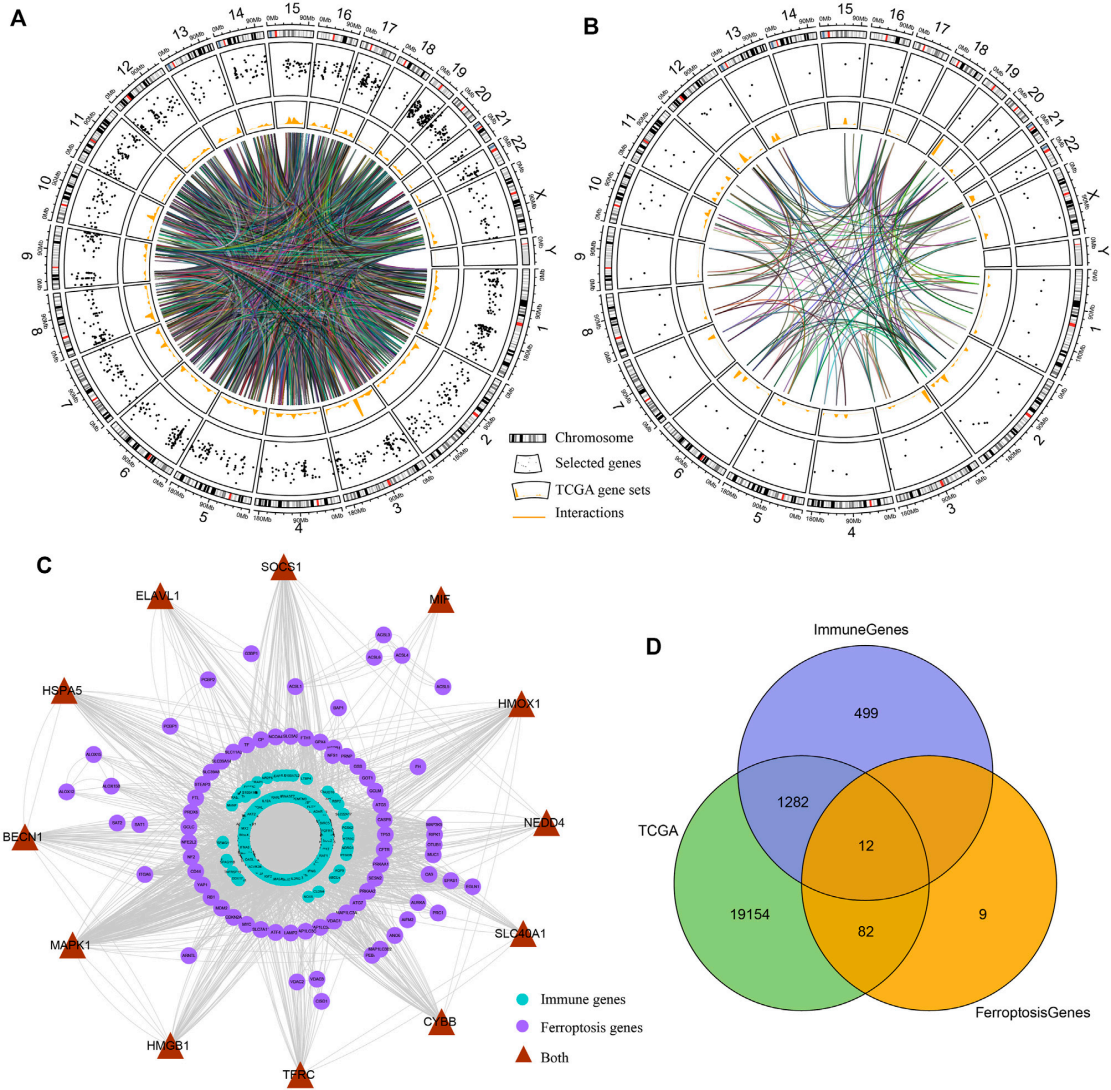
Interactions between immune genes and ferroptosis-related genes. **(A,B)** Circos plots illustrating the annotation and cross-talk of immune genes and ferroptosis-linked genes, respectively, in the genome of TCGA dataset. Outer circle illustrates individual genes’ positions on chromosomes. Scatter plots in the second circle designate the genes. Third circle demonstrates the relative levels of expressions of the genes in TCGA cohort. Central lines designate the possible cross-talks between genes forecasted by the STRING data resource. **(C)** PPI network of immune genes and ferroptosis-linked genes predicted by the STRING database. Purple nodes designate ferroptosis-linked genes, blue nodes indicate immune genes, and brown nodes are both ferroptosis-related and immune-related genes. **(D)** Venn diagram indicating ferroptosis-related and immune-related genes identified in TCGA cohort.

The transcriptome data in TCGA cohort were used to create a comprehensive indicator from immune- and ferroptosis-related profiling (Figures 1A,B). We constructed PPI networks of immune genes and ferroptosis-related genes. Most immune genes directly interacted with the ferroptosis-related genes according to the STRING database (Figure 1C). After data processing, 1294 immune genes and 94 ferroptosis-related genes were used for subsequent model construction (Figure 1D).

### Identification of Prognosis-Linked Signatures and Constructing the Prognostic Model

**FIGURE 2:**
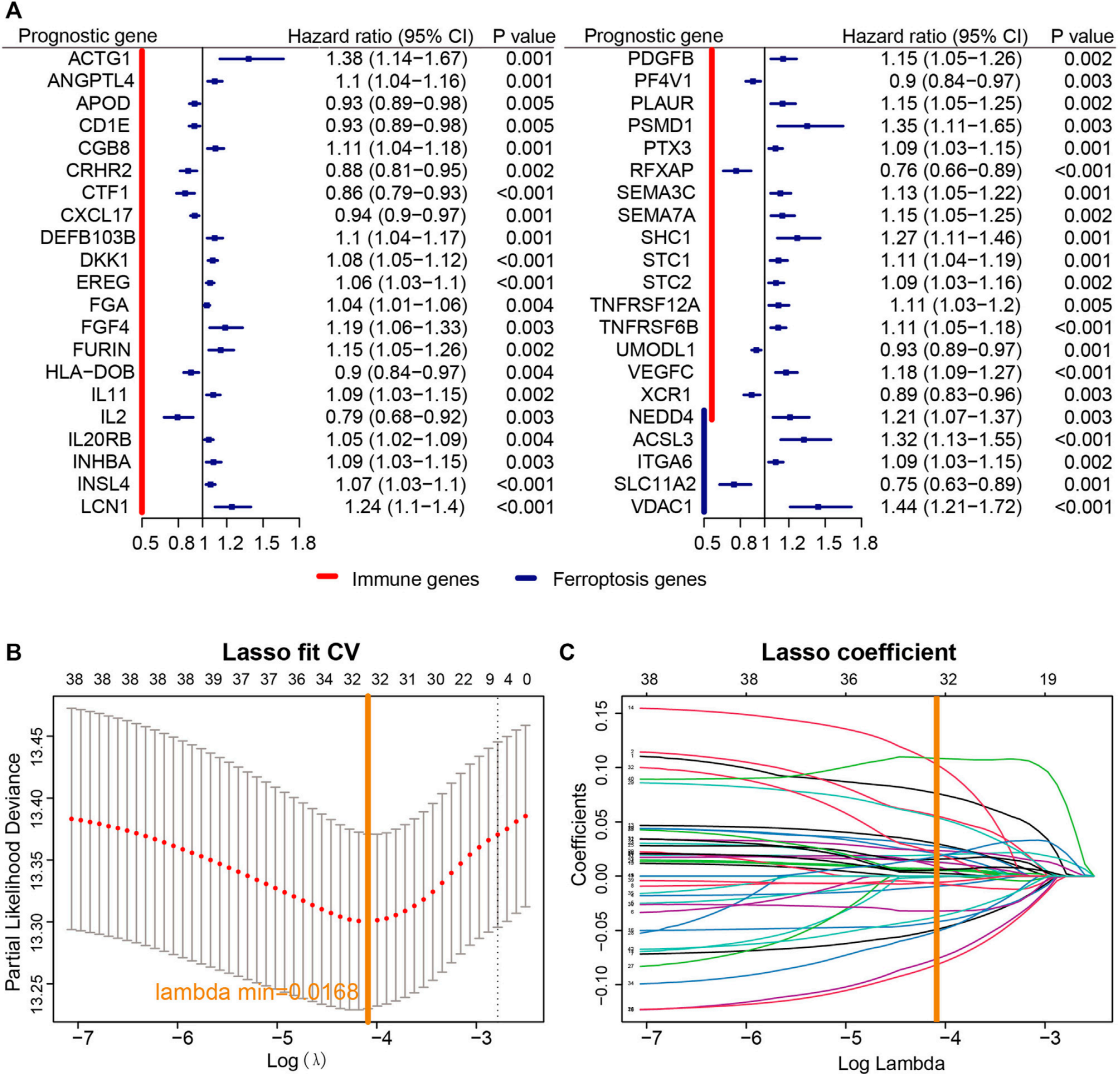
Establishment of the prognostic model. **(A)** According to univariate Cox proportional regression, 40 prognosis-related immune and ferroptosis-related genes were identified based on TCGA cohort. **(B)** Profiles of LASSO coefficients of 40 immune- and ferroptosis-linked genes. **(C)** Cross-confirmation for tuning selection of parameters in the LASSO model.

A univariate Cox proportional regression model was used to explore the prognostic value of both immune- and ferroptosis-linked genes. Screened with *p* < 0.05, 42 prognosis-related genes were obtained (Figure 2A), including 38 immune-related genes and five ferroptosis-related genes, among which, *NEDD4* was both an immune- and ferroptosis-related gene (Figure 2A). *NEDD4* is an oncogene, which encodes E3 ubiquitin ligase. Next, a LASSO analysis was used to construct a prognostic model with 32 signatures: *ACSL3*, *ACTG1*, *ANGPTL4*, *APOD*, *CD1E*, *CRHR2*, *CTF1*, *DEFB103B*, *DKK1*, *EREG*, *FGA*, *FGF4*, *HLA-DOB*, *IL2*, *INSL4*, *ITGA6*, *LCN1*, *NEDD4*, *PDGFB*, *PF4V1*, *PTX3*, *RFXAP*, *SEMA3C*, *SEMA7A*, *SHC1*, *SLC11A2*, *STC2*, *TNFRSF6B*, *UMODL1*, *VDAC1*, *VEGFC,* and *XCR1* (Figures 2B,C). Then, based on the expression of the optimized 32 signatures and correlation in TCGA cohort, we established a predictive model.

### Verification of the Prognostic Model

**FIGURE 3:**
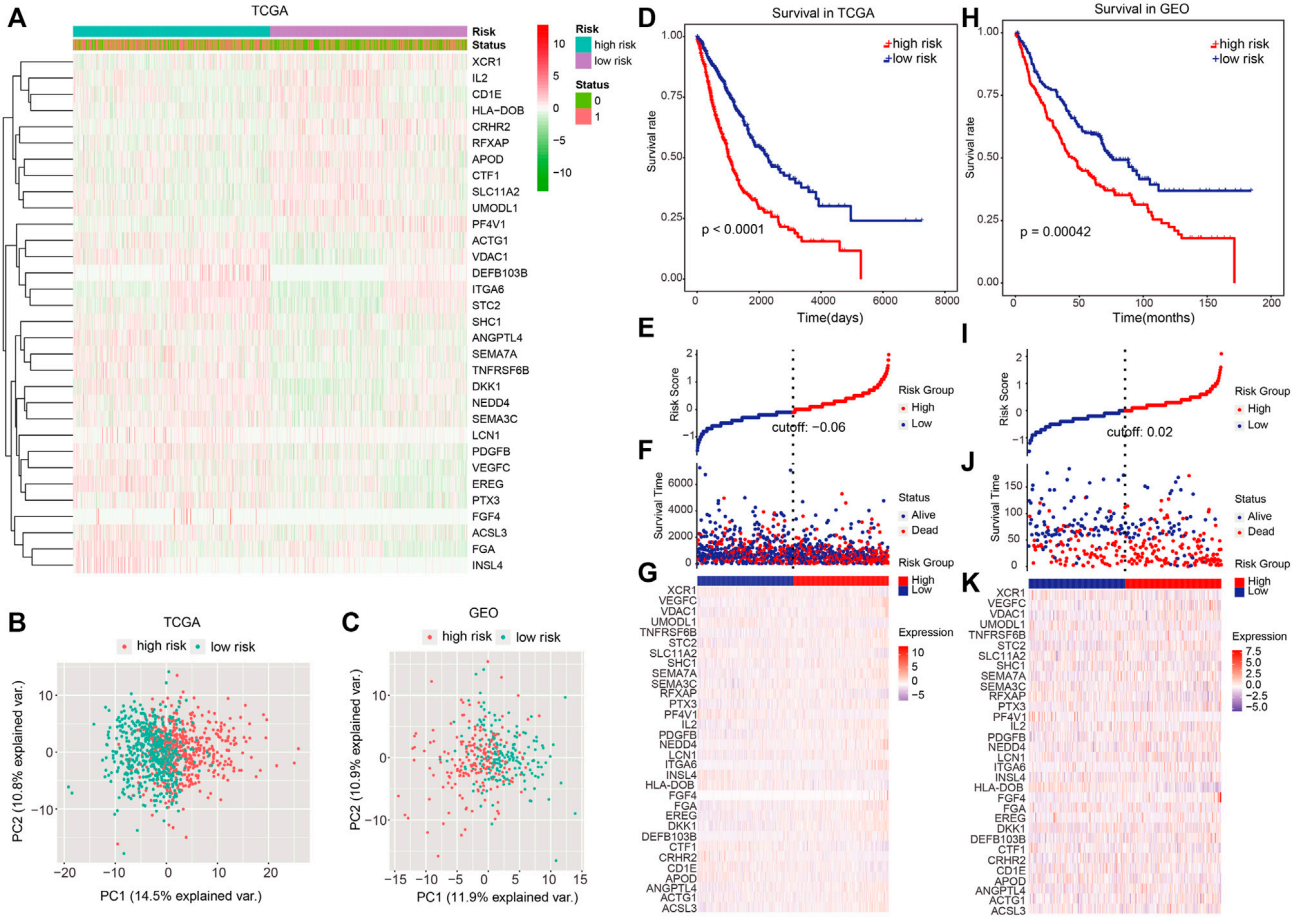
Dividing power of prognostic models. **(A)** Heatmap showed the expression of 32 signatures we used for constructing the model. **(B,C)** Principal component analysis for TCGA and GEO cohorts based on the expression of the 32 signatures. **(D)** Kaplan–Meier plot was adopted to show the difference in OS between high- and low-risk groups in TCGA dataset. **(E)–(G)** Survival time and risk score distributions on the basis of the prognostic model in TCGA dataset. **(H)** Difference in OS between high- and low-risk groups in the independent validation set (GEO cohort). **(I–K)** Survival time, as well as risk score distributions in the GEO cohort. *p* < 0.05 denoted statistical significance.

Every patient’ risk score in TCGA and GEO data sets was computed. For analyzing the accuracy of the signatures used for constructing the module, we visualized the expression of 32 genes and found that a majority of 32 genes were differentially expressed between the high-risk and low-risk groups (Figure 3A). Then, PCA was performed to investigate whether lung cancer patients could be distinguished according to the expression of the 32 signatures in TCGA and GEO data sets (Figures 3B,C).

Next, patients were categorized into the high- and low-risk group using the median risk score as the cutoff value. In TCGA data set, patients in the high-risk group had a remarkably worse OS (Figure 3D; *p* < 0.0001; log-rank test), and the number of alive patients in the low-risk group were more relative to those in the high-risk group (Figures 3E–G). In the independent validation set (GEO dataset), patients in the high-risk group also exhibited a remarkably worse OS (Figure 3H; *p* = 0.00042 log-rank test), and the alive patients in low-risk group were more than those in the high-risk group (Figures 3I–K).

### Risk Score Connected With Clinical Pathological Characteristics

**FIGURE 4:**
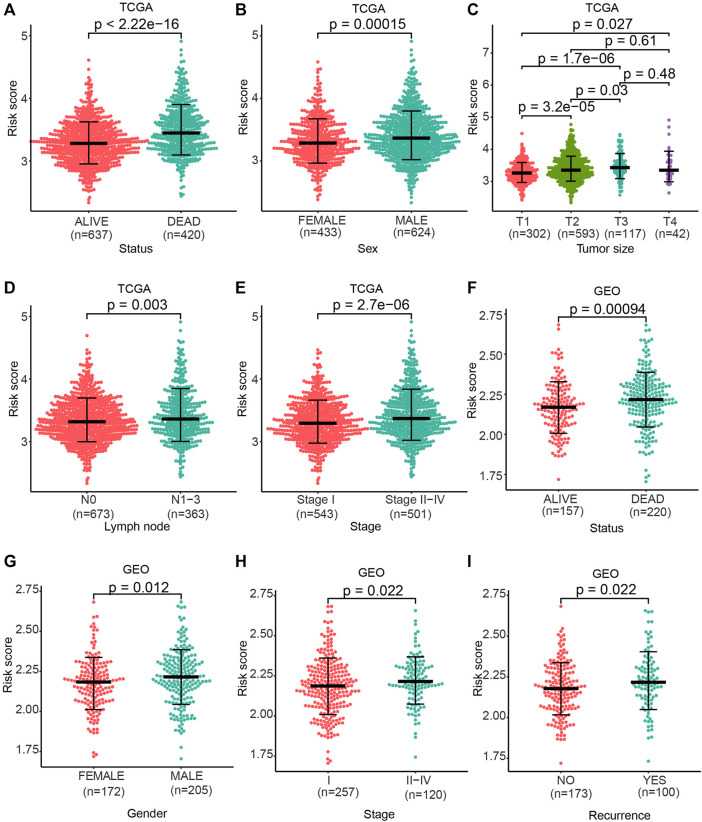
Risk score discrepancy between the subgroup of clinical characteristics. **(A–E)** One-sided Wilcoxon rank sum test was used to evaluate the differences in the risk score between patient status groups, sex groups, tumor size groups, lymph node groups, and pathological stage groups in TCGA cohort. **(F–I)** One-sided Wilcoxon rank sum test was used to evaluate the differences in the risk score between patient status groups, sex groups, pathological stage groups, and recurrence groups in the GEO cohort. *p* < 0.05 denoted statistical significance.

We next explored the capacity of the prognostic model in clinical pathological characteristics. We first investigated the difference of clinical characteristics between the high-risk and low-risk groups (Table 1). Then, we evaluated the differences of the risk score between patients with different clinical characteristics (dead *vs*. alive), sex groups (female *vs*. male), tumor size groups (T1 *vs*. T2 *vs*. T3 *vs*. T4), lymph node (N0 *vs*. N1-N3), and pathological stage (Stage I *vs*. Stage II-Stage IV) in TCGA cohort. The risk score in dead patients was remarkably higher in contrast with alive patients (Figure 4A; *p* < 2.22e-16), and male patients were remarkably higher than female patients (Figure 4B; *p* = 0.00015). The risk score in stage T2, T3, and T4 was remarkably higher relative to stage T1, and the risk score in stage T3 was remarkably higher than stage T2 (Figure 4C; *p* < 0.05). Besides, the risk score in stage N1–N3 was remarkably higher in contrast with stage N0 (Figure 4D; *p* = 0.003), and stage II–stage IV was remarkably higher than stage I (Figure 4E; *p* = 2.7e-06). Moreover, the risk score of dead patients was remarkably higher relative to alive patients (Figure 4F; *p* = 0.00094), and male patients were remarkably higher than female patients (Figure 4G; *p* = 0.012). Stage II–Stage IV was remarkably higher than stage I (Figure 4H; *p* = 0.022), and recurrent patients were remarkably higher than non-recurrence patients (Figure 4I; *p* = 0.022) in the GEO cohort.

**FIGURE 5:**
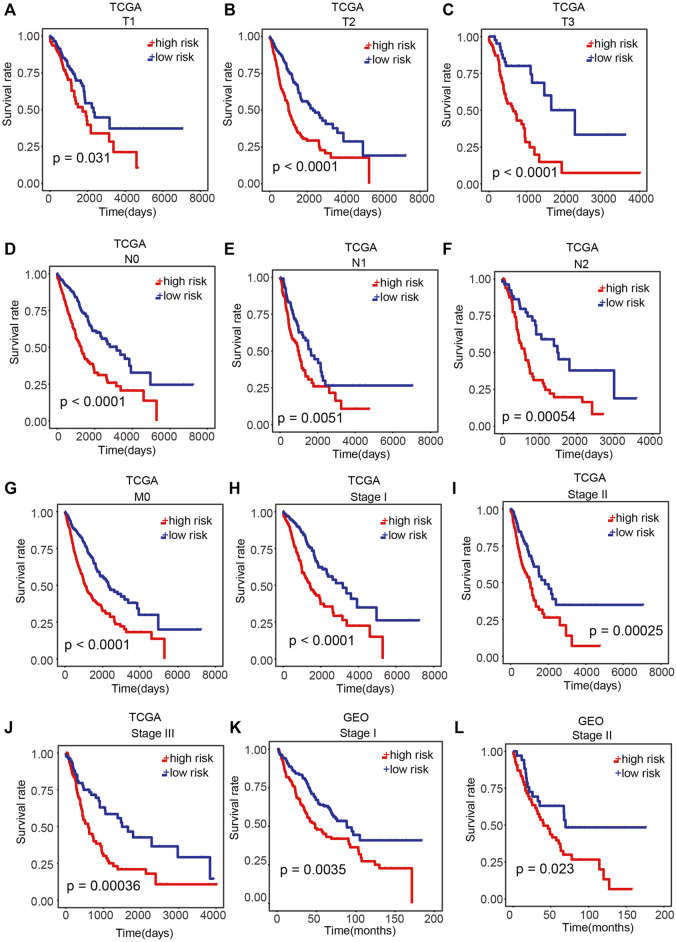
Performance of our prognostic model in patients with different clinical characteristics. **(A–J)** Difference in OS between the high-risk and low-risk samples of T subgroups (T1, T2, and T3), N subgroups (N0, N1, and N2), M0 subgroups, and pathological stage (stage I, stage II, and stage III) of TCGA cohort. **(K,L)** Difference in OS between high-risk and low-risk samples in pathological stage (stage I and stage II) of the GEO cohort. *p* < 0.05 was regarded remarkable.

Further assessment was conducted to explore whether the risk score reveals prognosis in diverse subgroups of clinical features. In the T subgroups (T1, T2, and T3), N subgroups (N0, N1, and N2), M0 subgroup, and pathological stage (stage I, stage II, and stage III) of TCGA cohort, patients in the high-risk group exhibited a poor OS (Figures 5A–J; *p* < 0.05; log-rank test). In the pathological stage (stage I and stage II) of the GEO cohort, remarkably poorer OS was found in patients in the high-risk group (Figures 5K,L; *p* < 0.05; log-rank test).

A multivariable Cox proportional regression model was built in TCGA cohort using the risk score and clinical pathological stage groups to verify the prognostic potential and independence of the prognostic model from other clinico-pathologic characteristics. The result suggested that our prognostic model has a potential in clinical application (Table 2).

The discrepancy of immune infiltration and ferroptosis between different risk groups.

**FIGURE 6:**
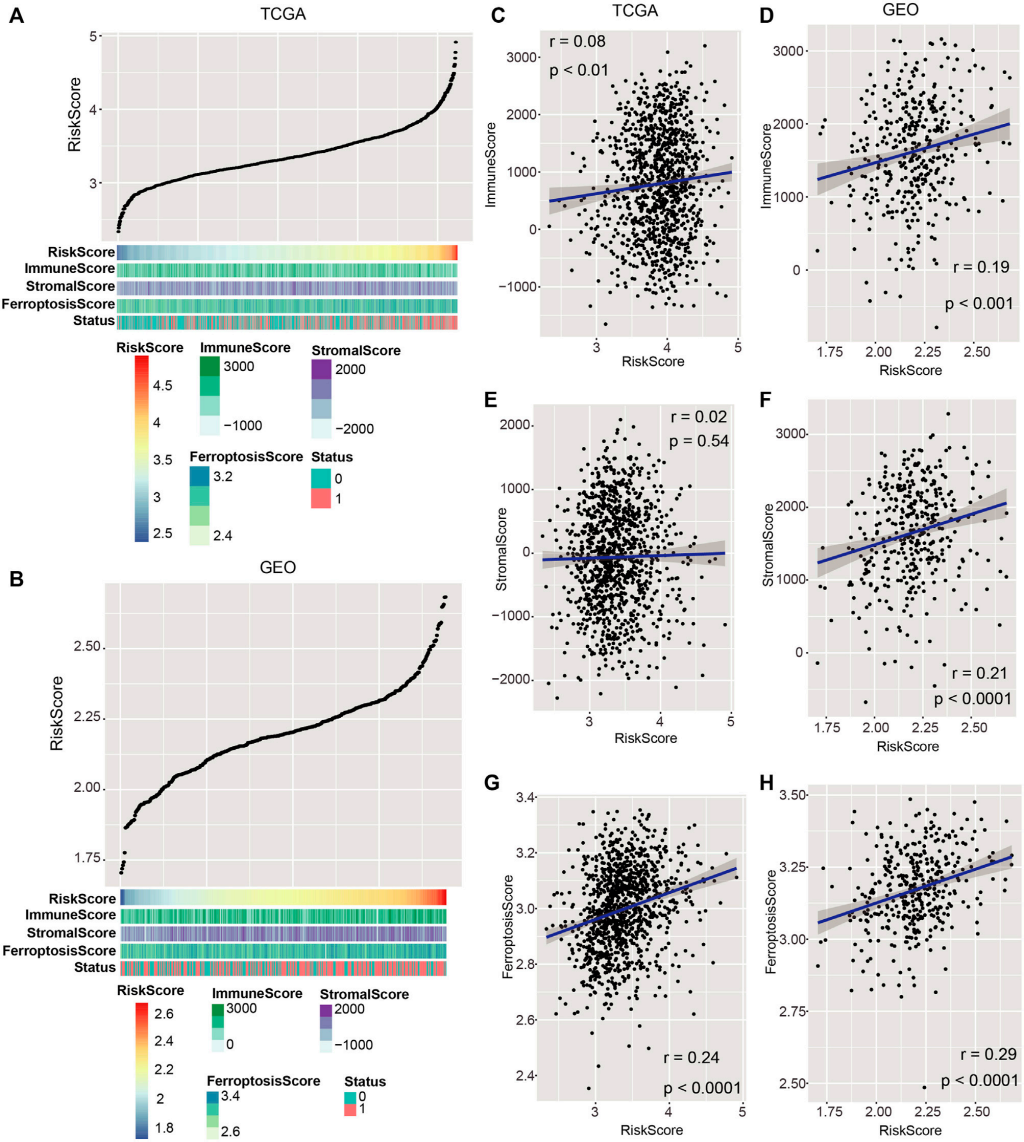
Associations of the risk score with immune invasion and ferroptosis. **(A,B)** Distribution of Immune Score, Stromal Score, and Ferroptosis Score with the increase of risk score in both TCGA and GEO datasets. **(C,H)** Pearson’s correlation analysis was proceeded to discern the relation of Immune Score, Stromal Score, and Ferroptosis Score with the risk score in both TCGA and GEO cohorts. *p* < 0.05 was regarded remarkable.

To investigate the relationship between the risk score and immune infiltration as well as ferroptosis, we analyzed the distribution of the ESTIMATE score (consists of Immune Score and Stromal Score) and Ferroptosis Score (enrichment score of ferroptosis-related genes) in each sample. Immune Score, Stromal Score, and Ferroptosis Score tended to increase with the escalation of the risk score in both TCGA and GEO cohorts (Figures 6A,B). To further confirm this trend, Pearson’s correlation analysis was calculated between the risk score and Immune Score, Stromal Score, and Ferroptosis Score, respectively. The results showed that they were positively related with the risk score in both TCGA and GEO cohorts (Figures 6C–H; *p* < 0.05; Pearson’s correlation analysis).

**FIGURE 7:**
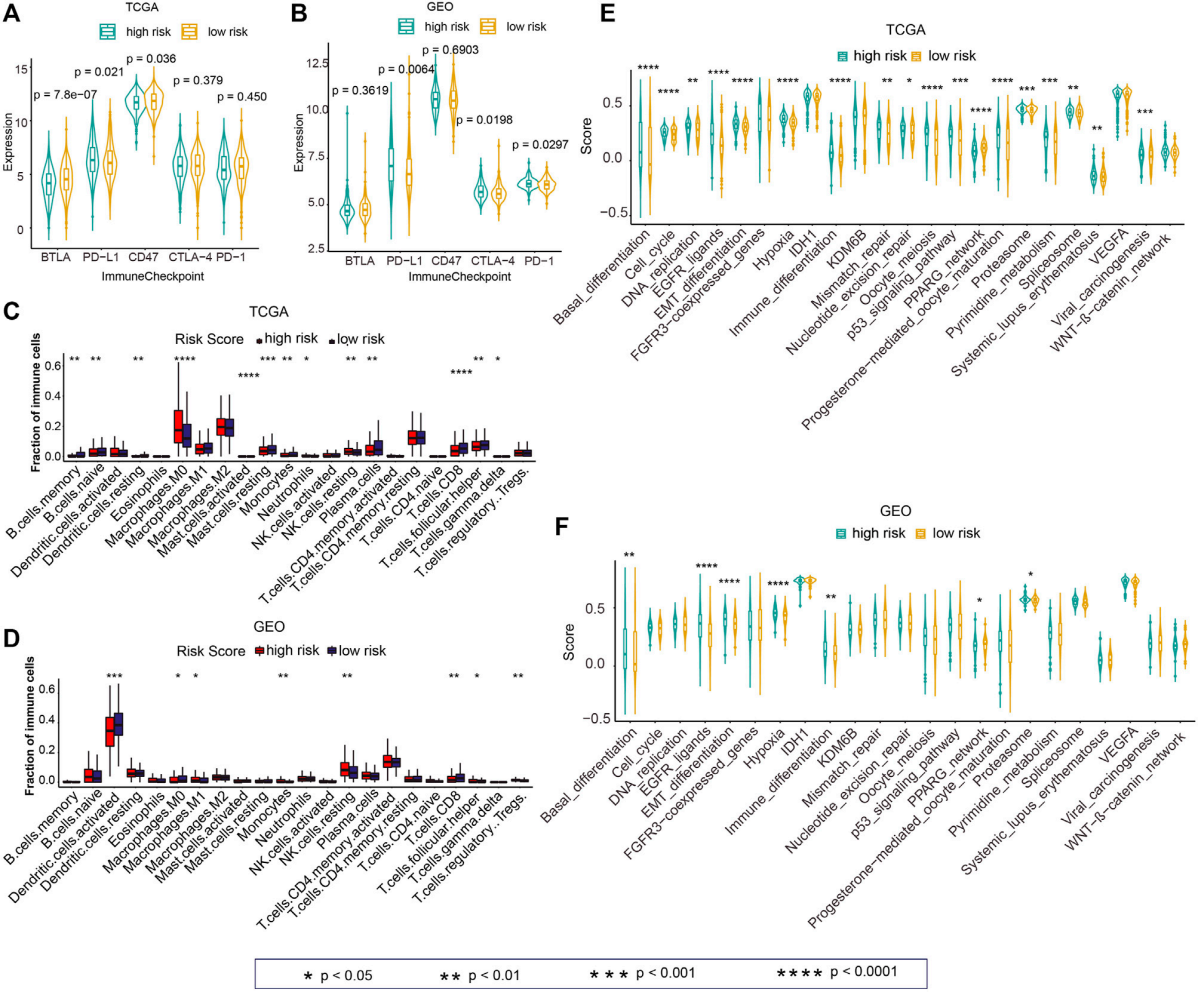
Associations of the risk score with the tumor immune microenvironment and cancer therapeutic score. **(A,B)** Differences in the expressions of five immune checkpoint–linked genes between high- and low-risk groups in TCGA and GEO cohorts. **(C,D)** Differences in 22 immune cell infiltration between high- and low-risk groups in TCGA and GEO datasets. **(E,F)** Differences in the cancer therapeutic enrichment score in high- and low-risk groups in TCGA and GEO datasets. *p* < 0.05 was regarded statistically significant.

Next, we investigated the difference in the expression of five immune checkpoint–linked genes between high- and low-risk groups. The expression of PD-L1 in the high-risk group was remarkably greater than that in the low-risk group in TCGA and GEO cohorts (Figures 7A,B; *p* = 0.021, *p* = 0.0064). Besides, CTLA-4 and PD-1 expressions in the high-risk group were greater than those in the low-risk group in the GEO cohort (Figure 7B; *p* = 0.0198, *p* = 0.0297).

To explore immune cell infiltration of tumor samples, we calculated 22 immune cell abundances among TCGA and GEO cohorts by CIBERSORT. Next, we explored the difference of 22 immune cell invasion between high- and low-risk groups. The invasion of M0 Macrophages in the high-risk group was remarkably greater in contrast with that in the low-risk group in TCGA cohort (Figure 7C; *p* < 0.0001). The infiltration of resting NK cells in the high-risk group was remarkably higher than that in the low risk group in TCGA and GEO cohorts (Figures 7C,D; *p* < 0.01). Also, significantly greater infiltration of CD8 T cells was observed in the high-risk group relative to that in the low-risk group in TCGA and GEO data sets (Figures 7C,D; *p* < 0.01).

We next investigated the potential role of the prognostic model in the prediction of response to immunotherapy using the IMvigor210 cohort. We found that the risk score in non-responsive patients [stable disease (SD) and progressive disease (PD)] was significantly higher than responsive patients [complete response (CR) and partial response (PR)] (Supplementary Figure S1A; *p* < 0.05; one-sided Wilcoxon rank sum test). The number of non-responsive patients in the high-risk group was more than that in the low-risk group (Supplementary Figure S1B). The distribution of CR, PR, SD, and PD patients between the high-risk and low-risk groups was significant (Supplementary Figure S1C; *p* < 0.05; chi-square test). Besides, patients in the high-risk group had significantly poorer OS (Supplementary Figure S1D; *p* < 0.01).

### Correlation of Risk Score and Cancer Therapeutic Potential

For investigating the guiding role of the risk score in cancer treatment, we calculated the enrichment score for each sample according to 23 cancer therapeutic–predicted signature sets by ssGSEA. Next, we analyzed the differences of these scores in the high-risk and low-risk groups in TCGA and GEO cohorts. In TCGA cohort, the enrichment score of the high-risk group was higher relative to the low-risk group in 73.91% (17/23) of cancer therapeutic prediction signature sets, including “Basal_differentiation”, “Cell_cycle”, “DNA_replication”, “EGFR_ligands”, “EMT_differentiation”, “Hypoxia”, “Immune_differentiation”, “Mismatch_repair”, “Nucleotide_excision_repair”, “Oocyte_meiosis”, “p53_signaling_casacde”, “Progesterone-mediated_oocyte_maturation”, “Proteasome”, “Spliceosome”, “Pyrimidine_metabolism”, “Systemic_lupus_erythematosus”, and “Viral_carcinogenesis” (Figure 7E; *p* < 0.05). In the GEO cohort, the elevated score of “Basal_differentiation”, “EGFR_ligands”, “EMT_differentiation”, “Hypoxia”, “Immune_differentiation”, and “Proteasome” was observed in the high-risk group in contrast with those in the low-risk group (Figure 7F; *p* < 0.05).

## Discussion

Immune status and ferroptosis are both important in NSCLC. Tumor immune microenvironment–related signature could estimate the prognosis of NSCLC patients, which may also be indicators for immunotherapy (Ojlert et al., 2019; Li et al., 2020). Recently, a ferroptosis-linked gene-based prognostic model was constructed by Han et al. They found that the ferroptosis-related risk score was linked to immune status (Han et al., 2021). Although clinical indicators regarding immune response and ferroptosis have been established, few investigations focused on their combined effect and their clinical application capacity have been performed. Herein, we explored the potential role of a combined immune and ferroptosis model for NSCLC.

Gene expression data were obtained from TCGA and GEO databases, which served as training and testing datasets, respectively. Immune- and ferroptosis-related genes were identified through databases and publications. After data processing, we collected 1294 immune genes and 94 ferroptosis-related genes (Figure 1).

A univariate Cox proportional regression model was used to identify immune- and ferroptosis-linked genes that have prognostic potential of NSCLC in the TCGA dataset. Overall, genes were analyzed, including 1294 immune-related genes and 94 ferroptosis-related genes, and 12 of these genes are related to both immune response and ferroptosis. Screened with *p* < 0.05, 42 prognosis-related genes were obtained, including 38 immune-related genes and 5 ferroptosis-related genes, among which, *NEDD4* was both an immune- and ferroptosis-related gene (Figure 2). *NEDD4* is an oncogene, which encodes E3 ubiquitin ligase. *NEDD4* is remarkably correlated with the migration of NSCLC cells (Shao et al., 2018). Knockdown of *NEDD4* could inhibit the migration of NSCLC cells (Shao et al., 2018). *NEDD4* is also related to drug resistance of NSCLC cells. The downregulation of *NEDD4* could elevate the effect of afatinib in afatinib-resistant H1975 clones (Booth et al., 2018). *NEDD4* was also associated with the erlotinib resistance of NSCLC by inhibiting *PTEN* expression (Sun et al., 2017). Moreover, *NEDD4* could be the therapeutic target for NSCLC. The anticancerous effect of nitidine chloride was evaluated through the inhibition of *NEDD4* in NSCLC H1299 cells, which was abrogated by the overexpression of *NEDD4* (Zhang et al., 2020).

Of these 42 genes, 32 of them were selected to compute the risk of NSCLC. On the basis of the LASSO Cox regression model, the samples were stratified into high-risk and low-risk groups. Then, we analyzed the OS in TCGA and GEO cohorts. Patients in the low-risk group have better OS in contrast with those in the high-risk group in both the cohorts (Figure 3).

We then evaluated the differences of the risk score among the clinical pathological subgroups. The higher risk score was observed in dead samples, larger tumor size, higher cancer stage, and recurrence cohorts, respectively (Figure 4). Especially, we found that the risk score was lower in early stages than in later stages, but there was no difference in each stage (Figure 4; Table 1). The prognosis potential of the risk score was investigated in different subgroups and pathological stages of NSCLC. The results showed that patients exhibiting a high risk score have shorter OS in all subgroups (T, N, and M0 subgroups) and pathological stages (stage I, II, and III) (Figure 5).

Subsequently, we assessed the relationship of the risk score and immune invasion with ferroptosis. Our developed risk score was found to be positively correlated with Immune Score, Stromal Score, and Ferroptosis Score in TCGA and GEO cohorts (Figure 6). This result was in accordance with the previous findings that Immune Score, Stromal Score, and Ferroptosis Score were all prognosis indicators for cancer (Shen et al., 2019; Wang H. et al., 2019; Liang et al., 2020). The relationship of the risk score with the immune checkpoint–linked genes (BTLA, PD-L1, CD47, CTLA-4, and PD-1) was evaluated. PD-L1 expression was elevated in the high-risk score group relative to that in the low-risk score group in both cohorts (Figures 7A,B). It is widely accepted that immunotherapies are effective for NSCLC patients with high PD-L1 expression. Regardless of histologic type, atezolizumab treatment remarkably prolonged the OS of NSCLC with high PD-L1 expression than platinum-based chemotherapy (Herbst et al., 2020). NSCLC harboring EGFR mutations exhibited an immune-inert phenotype, which was characterized by low expression of PD-L1, low tumor mutational burden, low cytotoxic T-cell number, and low T-cell receptor clonality. This kind of NSCLC lacks clinical response to immune checkpoint blockade therapy (Le et al., 2021).

Next, the immune cell infiltration between the high-risk and the low-risk groups was analyzed. The infiltration of CD8^+^ T cells was lower in the high-risk group in contrast with the low-risk group in TCGA along with GEO cohorts (Figures 7C,D). CD8^+^ T-cell infiltration is considered an independent predictive factor for NSCLC (Donnem et al., 2015). Hurkmans et al. suggested that the combination of PD-L1 expression, TML, CD8^+^ T-cell infiltration, and HLA class-I functions could be used to predict the efficiency of immunotherapy in NSCLC patients (Hurkmans et al., 2020). Combined with the results of Figures 7A,B, in which PD-L1 expression was higher in the high-risk score group than in the low-risk score group, we concluded that our prognostic model integrating ferroptosis and immune infiltration could be used as a potentially predictive biomarker for response to immunotherapy. Furthermore, we investigated the potential role of the prognostic model in the prediction of response to immunotherapy using the IMvigor210 cohort. We found that the risk score in non-responsive patients was significantly higher than in responsive patients (Supplementary Figure S1A) and the patients in the high-risk group had significantly poorer OS (Supplementary Figure S1D), suggesting the potential use of the prognostic model in immunotherapy.

Finally, the guiding role of the risk score in cancer treatment was evaluated. “EGFR_ligands”, “EMT_differentiation” “Hypoxia”, “Immune_differentiation”, and “Proteasome” were positively associated with the risk score in TCGA and GEO cohorts. These factors are all important prognosis biomarkers and therapeutic targets for NSCLC. For example, hypoxia is linked to poor prognosis and could induce resistance of NSCLC (Salem et al., 2018; Shi et al., 2019; Hua et al., 2020; Lu et al., 2020). EGFR and proteasomes play a pivotal role in NSCLC development, and their inhibitors could be used in NSCLC treatment (Li et al., 2009; Liu et al., 2014; Floc’h et al., 2018; Tanimoto et al., 2021). Whereas, “PPARG_network” was inversely related with the risk score in TCGA and GEO datasets. It is reported that PPARG c.1347C>T polymorphism was correlated with the risk of NSCLC (Ding et al., 2017). PPARG was downregulated in NSCLC samples, and the enhanced expression of PPARG may inhibit the development and progression of NSCLC (Shi et al., 2020).

## Conclusion

In conclusion, by analyzing a total of 1376 immune- and/or ferroptosis-related genes, we developed a ferroptosis and immune-combined index with 32 genes for NSCLC prognosis. The integrated predictor may help distinguish the heterogeneity of NSCLC and effectively improve the prognostic value. However, the study cohorts we used only included LUAD and LUSC. This limitation will be greatly alleviated by the development of cancer big-data. Also, sufficient experimental verification is needed to explore the potential mechanisms of ferroptosis in NSCLC.

## Data Availability

The datasets presented in this study can be found in online repositories. The names of the repository/repositories and accession number(s) can be found in the article/Supplementary Material.

## Supplementary Material

SUPPLEMENTARY FIGURE 1 Predictive value of the risk score in immunotherapy response. **(A)** Distribution of the risk score between responsive patients [complete response (CR) and partial response (PR)] and non-responsive patients [stable disease (SD) and progressive disease (PD)] in the IMvigor210 cohort. *P* value was provided by the one-sided Wilcoxon rank sum test. **(B)** Distribution of responsive and non-responsive patients between the high-risk and low-risk groups. *P-*value was provided by the chi-square test. **(C)** Distribution of CR, PR, SD, and PD patients between the high-risk and low-risk groups. *P-value* was calculated based on the chi-square test. **(D)** Kaplan–Meier curves of high-risk and low-risk groups in the IMvigor210 cohort. *P-value* was provided based on the log-rank test.
