# Supplementary figures and images for: Identification and Validation of an Immune and Ferroptosis-Combined Index for Non–Small Cell Lung Cancer
Source: Front Genet. 2021 Nov 30;12:764869. doi: 10.3389/fgene.2021.764869 (PMC8669617; doi:10.3389/fgene.2021.764869)

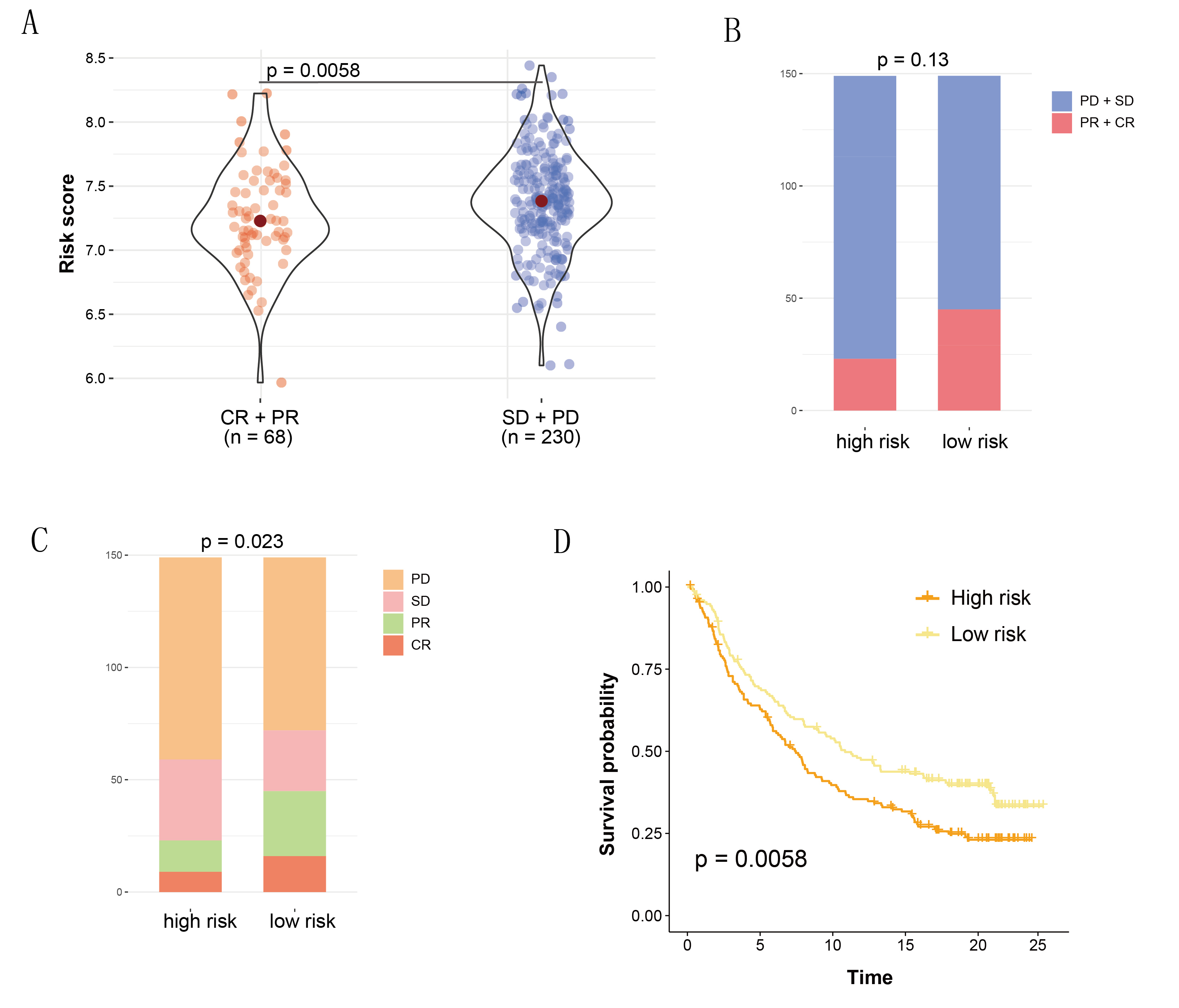

Supplement: Supplementary file 1 [file Image1.JPEG]
